# Left Transperitoneal Adrenalectomy with a Laparoendoscopic Single-Site Surgery Combined Technique: Initial Case Reports

**DOI:** 10.1155/2011/651380

**Published:** 2011-04-14

**Authors:** Yasuhiro Sumino, Daisaku Nakano, Ken-Ichi Mori, Takeo Nomura, Fuminori Sato, Hiromitsu Mimata

**Affiliations:** Department of Urology, Faculty of Medicine, Oita University, Idaigaoka 1-1, Yufu City, Oita 879-5593, Japan

## Abstract

Laparoendoscopic single-site surgery (LESS) is a step toward the development of minimally invasive surgery. It is initially difficult for surgeons with limited experience to perform the surgery. We describe two cases of left adrenalectomy with a LESS combined with the addition of an accessory port. After a 2.5-cm skin incision was made at the level of the paraumbilicus to insert the primary 12-mm trocar for the laparoscope, a 5-mm nonbladed trocar was placed through the skin incision side-by-side with the primary trocar. A second 3-mm nonbladed trocar was then placed along the anterior axillary line; a multichannel trocar was not used as a single port. Both adrenalectomies were completed successfully. In patients with a minor adrenal tumor, a combined technique using LESS and an additional port is easier than LESS alone and may, therefore, be a bridge between the conventional laparoscopic approach and LESS.

## 1. Introduction

Laparoendoscopic single-site surgery (LESS) is a step toward the development of minimally invasive surgery [1–5]. However, LESS may be initially difficult for surgeons who have limited experience with it, even if they are experienced in traditional laparoscopic surgery. 

We describe two cases of left adrenalectomy with a combined technique using LESS and the addition of an accessory port. This procedure is easy and safe for surgeons who have no experience with LESS. We, therefore, hope that it can be a bridge between the conventional laparoscopic approach and LESS.

## 2. Case Presentation

We experienced two cases of left adrenal tumor excised using a transperitoneal approach with a combined technique using LESS and an additional accessory port.

**Case 1**

**Figure 1:**
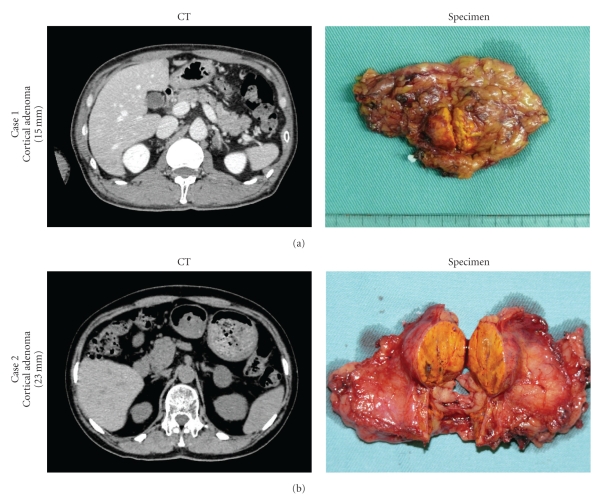
Preoperative CT scans and postoperative specimens of Cases 1 and 2, who underwent left adrenalectomy with combined technique using LESS with an additional accessory port.

A 54-year-old man (body mass index (BMI), 21.7) presented with hypertension and hypokalemia to our hospital. At examination, his serum aldosterone level was elevated, and computed tomography (CT) revealed a 15-mm left adrenal mass (Figure 1). According to the results of selective sampling of aldosterone through the venous circulation, he was diagnosed with primary aldosteronism. The operative time was 167 min (blood loss, 20 mL). A 6-mm tubular drain through the incision was left in situ. Postoperative recovery was uneventful. The patient started oral intake and was ambulatory on the first postoperative day and was discharged from the hospital on the eighth postoperative day. Pathologic examination confirmed cortical adenoma of the adrenal grand.

**Case 2**

A 69-year-old woman (BMI, 24.4) with a diagnosis of hypertension presented to our hospital. Her 24-h urine catecholamine levels were slightly elevated, and CT scan revealed a 23-mm left adrenal mass (Figure 1). I^131^-metaiodobenzylguanidine scintigraphy did not reveal a functional lesion around the left adrenal grand. The patient was diagnosed with suspected pheochromocytoma and was given 4 mg of doxazosin to correct her circulating plasma volume. The operative time was 165 min (blood loss, 20 mL). A 6-mm tubular drain was not left in place in this case. Postoperative recovery was uneventful, as with Case 1. The patient started oral intake and was ambulatory on the first postoperative day and was discharged from the hospital on the tenth postoperative day. Pathologic examination confirmed cortical adenoma of the adrenal grand.

Both patients required analgesic agents on the first postoperative day but not during discharge.

## 3. Surgical Technique

**Figure 2:**
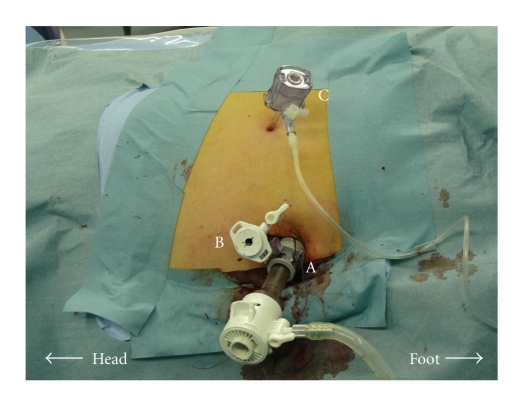
Trocar placement for left adrenalectomy with the LESS combined technique. The primary 12-mm trocar (A) and a 5-mm nonbladed trocar (B) were located next to one another through a 2.5-cm skin incision. A 3-mm nonbladed trocar (C) was added along the anterior axillary line.

Surgery was performed with the patient at a 45° angle modified flank position under CO_2_ pneumoperitoneum and 12-cm H_2_O. No multichannel trocar was used as a single port. First, a 2.5-cm skin incision using the Hasson open technique was made at the level of the paraumbilicus for insertion of the primary 12-mm trocar for the laparoscope [6]. Second, a 5-mm nonbladed trocar was placed side-by-side with the primary trocar through the skin incision with endoscopic control. A 3-mm nonbladed trocar was then placed along the anterior axillary line; afterward, the abdomen was inspected (Figure 2).

**Figure 3:**
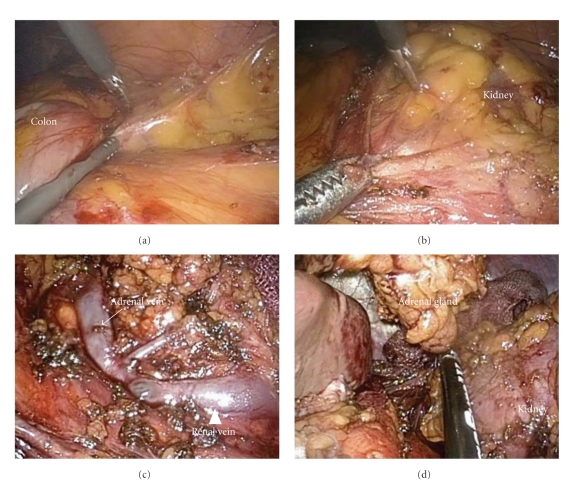
Intraoperative findings. (a) Incision of the white line of Toldt, (b) dissection of the junction of the colonic mesentery and Gerota's fascia, (c) renal vein (arrowhead) and left adrenal vein (arrow), and (d) adrenal dissection with a vessel-sealing device.

**Figure 4:**
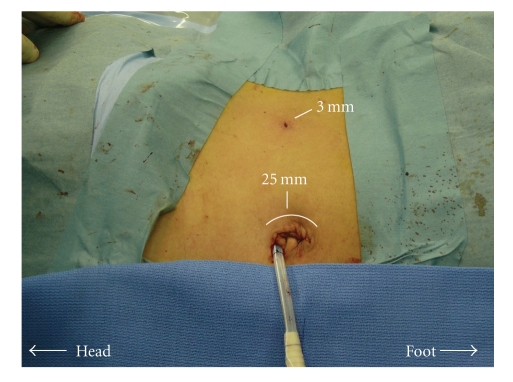
Postoperative appearance. A 6-mm tubular drain through the incision near the paraumbilicus was left in situ.

Using a flexible 5-mm laparoscope (Olympus, Japan), the peritoneal cavity was examined, with no unusual findings. The surgical strategy was conventional left transperitoneal adrenalectomy [7]. Once the white line of Toldt was incised using a combination of 3-mm monopolar scissors (held in the surgeon's right hand) and 5-mm monopolar forceps (held in the surgeon's left hand) (Figure 3(a)), the junction of the colonic mesentery and Gerota's fascia was identified (Figure 3(b)). This plane was then dissected to the renal vein (Figure 3(c)). The adrenal veins were identified, clipped with 5-mm Hem-O-Lok clips (Teleflex Medical, Research Triangle Park, NC, USA), held in the surgeon's left hand, and divided. A 5-mm vessel-sealing device (LigaSure V, COVIDIEN, CT, USA; held in the surgeon's left hand) was also used to complete the adrenal dissection (Figure 3(d)). The specimen was extracted by removing the 10-mm bag (Endo Catch Gold, COVIDIEN, CT, USA) through the enlarged paraumbilical trocar site by connecting the primary12- and 5-mm ports. Finally, the fascia and the skin were closed after a 6-mm tubular drain through the incision was left in situ (Figure 4).

## 4. Discussion

Traditional laparoscopic urologic surgery requires at least 3 or 4 ports and an additional incision to remove the specimen. On the other hand, natural orifice transluminal endoscopic surgery (NOTES) represents the ultimate in minimally invasive surgery by avoiding abdominal wall incision altogether [1, 2]. A procedure that minimizes these incisions as one larger incision is single-port (access and incision) laparoscopic surgery, also known as LESS.

Adrenalectomy with LESS is suitable in patients with a minor adrenal tumor, since the operative scar is small and therefore unnoticeable. Consequently, this procedure has gained acceptance as a suitable alternative to standard laparoscopic adrenalectomy for small adrenal masses [8, 9]. However, surgeons with limited experience in LESS may have difficulty secondary to the reduced triangulation, fog evacuation, and clashing of instruments. We have, therefore, described adrenalectomy with a combined technique using LESS with the addition of an accessory port and hope this will be a bridge between conventional laparoscopy and that with LESS approaches.

In our experience, adrenalectomy with the LESS combined technique is easy and safe. To perform adrenalectomy with LESS alone, the surgeon must be skilled in the use of the surgical instruments, which include articulable dissectors or scissors. However, the technique presented in the current study requires no instruments in addition to common equipment and materials (rigid scissors or vessel-sealing devices) readily available in the operating room, with the exception of a flexible laparoscope. Consequently, surgeons who have no experience with LESS could likely perform it easily, and this procedure could lead to the next step, which is pure LESS surgery.

In the current cases, we used a 12-mm trocar for the laparoscope despite using a flexible 5-mm laparoscope, since we originally assumed usage with a rigid laparoscope (about 10 mm in diameter). If we had selected a 5-mm trocar for a flexible 5-mm laparoscope from the beginning, the skin incision might have been shorter than 2.5 cm. Therefore, our procedure may have room for improvement.

In conclusion, adrenalectomy with the LESS combined technique is easier than that with LESS alone and could, therefore, be useful to surgeons who have no experience in LESS and may be considering using it in their practice. We, therefore, hope this procedure can be a bridge between the conventional laparoscopic approach and LESS.
